# The Impact of Electronic Health Records on the Duration of Patients’ Visits: Time and Motion Study

**DOI:** 10.2196/16502

**Published:** 2020-02-07

**Authors:** Abdulrahman Mohammed Jabour

**Affiliations:** 1 Department of Health Informatics Faculty of Public Health and Tropical Medicine Jazan University Jazan Saudi Arabia

**Keywords:** patients experience, time and motion, waiting time, electronic health records

## Abstract

**Background:**

Despite the many benefits of electronic health records (EHRs), studies have reported that EHR implementation could create unintended changes in the workflow if not studied and designed properly. These changes may impact the time patients spend on the various steps of their visits, such as the time spent in the waiting area and with a physician. The amount of time patients spend in the waiting area before consultation is often a strong predictor of patient satisfaction, willingness to come back for a return visit, and overall experience. The majority of prior studies that examined the impact of EHR systems on time focused on single aspects of patient visits or user (physicians or nurses) activities. The impact of EHR use on patients’ time spent during the different aspects of the visit is rarely investigated.

**Objective:**

This study aimed to evaluate the impact of EHR systems on the amount of time spent by patients on different tasks during their visit to primary health care (PHC) centers.

**Methods:**

A time and motion observational study was conducted at 4 PHC centers. The PHC centers were selected using stratified randomized sampling. Of the 4 PHC centers, 2 used an EHR system and 2 used a paper-based system. Each group had 1 center in a metropolitan area and another in a rural area. In addition, a longitudinal observation was conducted at one of the PHC centers after 1 year and again after 2 years of implementation. The analysis included descriptive statistics and group comparisons.

**Results:**

The results showed no significant difference in the amount of time spent by patients in the reception area (*P*=.26), in the waiting area (*P*=.57), consultation time (*P*=.08), and at the pharmacy (*P*=.28) between the EHR and paper based groups. However, there was a significant difference (*P*<.001) in the amount of time spent on all tasks between the PHC centers located in metropolitan and rural areas. The longitudinal observation also showed reduction in the registration time (from 5.5 [SD 3.5] min to 0.9 [SD 0.5] min), which could be attributed to the introduction of a Web-based booking system.

**Conclusions:**

The variation in the time patients spend at PHC centers is more likely to be attributed to the facility location than EHR use. The changes in the introduction of new tools and functions, however, such as the Web-based booking system, can impact the duration of patients’ visits.

## Introduction

Many studies have shown the benefits of electronic health records (EHRs) in reducing duplicate tests and procedures, reducing drug expenditures, improving the utilization of radiology tests, allowing for better documentation of charges, and decreasing billing errors [1-6]. On the other hand, some studies have reported that the new systems can disturb the current workflows and result in unintended consequences. The Agency for Healthcare Research and Quality defined workflow as “a sequence of physical and mental tasks performed by various people within and between work environments. It can occur at several levels (one person, between people, across organizations) and can occur sequentially or simultaneously” [1].

The patients’ waiting time and the consultation time are very important parts of patients’ experience that could be impacted by the introduction of EHR systems. Many studies have shown that physicians are concerned about the amount of time needed for data entry, and the physicians have stated that the data entry time could be better used to provide direct patient care [2-7]. The distribution of patients’ time during visits to primary health care (PHC) centers is a strong predictor of patient satisfaction and, thus, utilization. Studies have found that patients prefer to spend less time waiting for doctors, registering, or at the pharmacy and would prefer to have more time with physicians [8-10].

The vast majority of prior studies that investigated the impact of EHR on time can be categorized into two general classes of studies: efficiency studies and time and motion studies. Efficiency studies tend to focus on the number of patients who can be seen in a given period, whereas the majority of the EHR-related time and motion studies investigate the duration of a single task performed by health care providers [2-4,11-16]. Most patient-centered studies focused on the patient-physician interaction and the amount of time physicians allocate to patients. These studies examined the consultation time by comparing the time physicians allocate to EHR or electronic data entry with the amount of time physicians need for completing conventional paper-based documentation. Studies reported conflicting results regarding EHR’s effects on consultation time [11,15,17]. In addition, one study also reported that more variation was attributed to the facility location than the system being implemented [11].

The results of these studies provided details about patients’ experience and the amount of time spent at the doctor’s office but did not provide information about the time spent before or after a physician visit. Examples of time spent before and after a physician visit include the time spent in the waiting room before seeing a physician. To determine the impact of EHR on patients, it is important to investigate the impact of EHR from a patient’s perspective. The amount of time spent in a waiting area is strongly associated with patients’ satisfaction and willingness to revisit [8-10,18-20]. Similarly, other tasks that do not involve interactions with a physician impact patients’ satisfaction. These tasks could include registration and pharmacy services, which can add to the total duration of patients’ visit.

The duration of users’ experience with EHR can contribute to the duration of tasks at EHR-based facilities. Studies have indicated that user familiarity with a system is related to the amount of time per task. Some studies have highlighted reduced productivity in hospitals shortly after EHR implementation. The reduced productivity often improves as users become more familiar with the new system and develop the necessary skills to use the system efficiently. In some cases, the longer amount of time needed to perform tasks may continue, which can be explained by an EHR system having more functions and being more complicated than a comparable paper-based system [14]. The additional functions and features could result in a longer amount of time needed to complete tasks.

The aim of this study was to investigate the time patients spend at the various departments in PHC centers. The study focused on the following: time at registration, time spent in the waiting room, consultation time, and the time spent at the pharmacy. We hypothesized that the time patients spend at EHR-based and paper-based PHC centers is different. Furthermore, we hypothesized that the time patients spend at the EHR-based PHC centers will decrease with time after implementation.

## Methods

### Sites, Context, and Sampling

The Research Ethical Committee at Jazan University approved the study (approval number REC-39/4S005). We selected 4 PHC centers within Jazan area, Saudi Arabia, using a stratified randomized sampling. Of the 4 PHC centers, 2 used an EHR-based and 2 used a paper-based system. One of the 2 PHC centers using an her-based system was located in a metropolitan area and the other was located in a rural area. Similarly, 1 of the 2 PHC centers using a paper-based system was located in a metropolitan area and the other was located in a rural area.

Only public PHC centers operating under the Ministry of Health (MOH) were included in the study. These centers used the same policies and regulations related to funding, patient eligibility and coverage, and resources and were subject to the same laws. The MOH PHC centers provide public-free services to national citizens who make over 94% of the visitors, and the remaining noncitizens were covered through a private insurance or out-of-pocket [21]. Health care providers at the PHC centers included were general practitioners; some of the PHC centers provide basic dental services, which we excluded from this study.

As all PHC centers operate under the MOH, the 2 EHR-based PHC centers included were using the same EHR system, and the 2 paper-based PHC centers were using the same forms and documentation guidelines. Private and semipublic centers were excluded from the study to maintain homogeneity of sampling and to control for other confounding variables. More details about the PHC government solution strategy can be found on the Ministry of Health website [22].

### Observation and Data Collection

The data collected included both cross-sectional and longitudinal observations. To investigate the impact of familiarity and facility experience with EHR on the time patients spend at each phase of the visit, longitudinal data were collected. The longitudinal observation was carried out at 1 of the EHR-based PHC centers, first at baseline during January 2018 and then at follow-up during December 2018. The remaining 3 PHC centers were observed cross-sectionally during the same period of the longitudinal follow-up observation (during November 2018 and December 2018).

The observation was conducted by 8 undergraduate health informatics interns. To ensure the consistency of data collection, observers were trained for 2 weeks on the workflow concepts, observation, and the data collection techniques of the study. Before data collection, they also spent 3 days at each site to familiarize themselves with the workflow processes. On the fourth day, they began the collection of data via direct observation. The duration of each task was documented using a stopwatch and papers. The observers were also sharing their findings and experiences with the research team on a weekly basis during the observation period.

The study focused on the time spent on each task from a patient’s perspective and not the health provider’s perspective. For example, a patient’s waiting time is not a task that is based on the provider’s activity time. Moreover, because the emphasis was on the time spent from a patient’s perspective, the details of tasks or subtasks from a health care provider’s perspective were not differentiated. For example, from a health care provider’s perspective, patient registration involves the subtask of checking patient identities, entering patient information, and searching for the patient file. In this study, these tasks were considered as a single step—patient registration. In addition, if health care providers were performing parallel tasks, the duration of the tasks was measured as the time spent by the patient.

The definition of the beginning and end of each task was also defined from the patient’s perspective. The reception time was defined as the time from the beginning of patient-clerk encounter to the end of the registration process. The waiting time was defined as the time from the end of the reception time to the time patients entered the physician’s office. The consultation time was defined as the time from entering the physician’s office to the time exiting the office. The pharmacy time was defined as the time from the beginning of patient-pharmacist encounter to the time patients receive the medication and instruction. An important point to highlight is that all waiting (before consultation) takes place in the waiting room. Some studies showed that a physician may serve multiple rooms sequentially by inviting patients to one of the rooms, getting their vital signs taken by the nurse, and then having them wait for the physician in the same room. We found that each physician has a single room and patients were instructed to enter the main physician’s room when it was time to be seen.

For comparability, we also excluded the tasks that were not applicable to all PHC centers, such as the dental clinic or laboratory and x-ray. Patients who came for a dental visit or who end up going to the laboratory or x-ray department were excluded from the analysis. The reason for this is that patients who revisit the physician after an x-ray may have a different waiting time and consultation time from those visiting for an initial consultation.

### Analysis

Data were analyzed using the Statistical Package for the Social Sciences version 21 (IBM Corp). The analysis performed included descriptive statistics to show the time per task for the different groups.

For the group comparison, we performed the Mann-Whitney test, a nonparametric test. The comparison included the time patients spent at each phase of their visit at the PHC centers. The factors evaluated were EHR system versus paper-based system and metropolitan area versus rural area.

The Mann-Whitney test was also used to examine the impact of PHC familiarity with the EHR system and the time patients spent on each task during visits. This included comparing the time per task during the baseline and follow-up.

## Results

The results included comparing the time per task at the PHC centers using the EHR-based system with the PHC centers using the paper-based system and comparing the metropolitan PHC centers with the rural PHC centers. The results also included comparing the changes in the duration of tasks at 1 of the PHC centers after 1 year and after 2 years of using EHR.

### Electronic Health Record Versus Paper-Based Primary Health Care Centers

**Figure 1:**
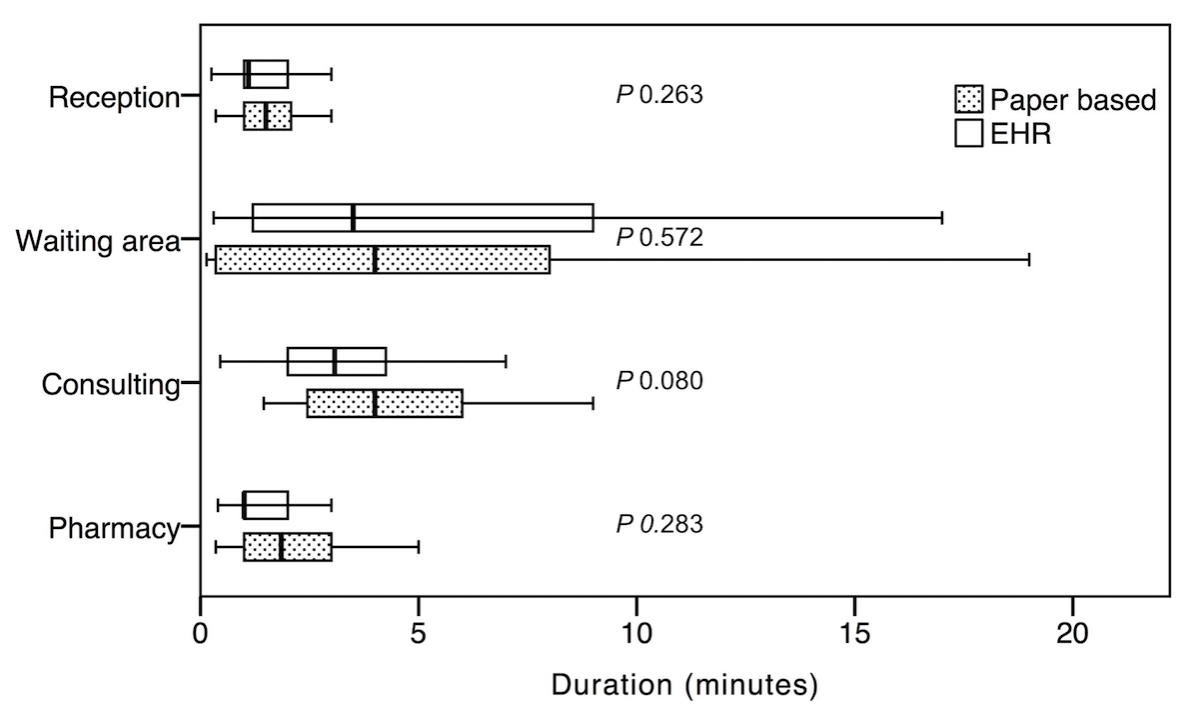
The time spent performing tasks at primary health care centers that use the electronic health record system and the paper-based system. EHR: electronic health record.

First, we observed the time spent performing basic tasks such as the registration time at the reception, time waiting for the physicians, consultation time, and time spent at the pharmacy. These observations were made at the 4 PHC centers. Other tasks such as getting x-rays and laboratory tests were excluded as these services were not available at all PHC centers. After the exclusion, the remaining number of events were 118 and 106 at the PHC centers using the EHR-based system and the PHC centers using the paper-based system, respectively. No significant differences were found between PHC centers that used an EHR-based system and those that used a paper-based system (*P*=.26, *P*=.57*, P*=.08, and *P*=.28 for the reception time, waiting time, consultation time, and time spent at the pharmacy, respectively; Table 1 and Figure 1).

**Table 1:** The time per task for primary health care centers using the electronic health record (EHR)–based system versus the paper-based system.

Task	EHR		Paper		*P* value
	Events, n	Time (min), mean (SD)	Events, n	Time (min), mean (SD)	
Reception	31	1.52 (1.02)	31	1.89 (1.36)	.26
Waiting for doctor	30	6.33 (7.37)	21	5.47 (6.11)	.57
Consultation time	28	3.30 (1.86)	26	6.39 (6.79)	.08
Pharmacy	29	1.61 (1.20)	28	1.95 (1.33)	.28

### Rural Versus Metropolitan Primary Health Care Centers

**Figure 2:**
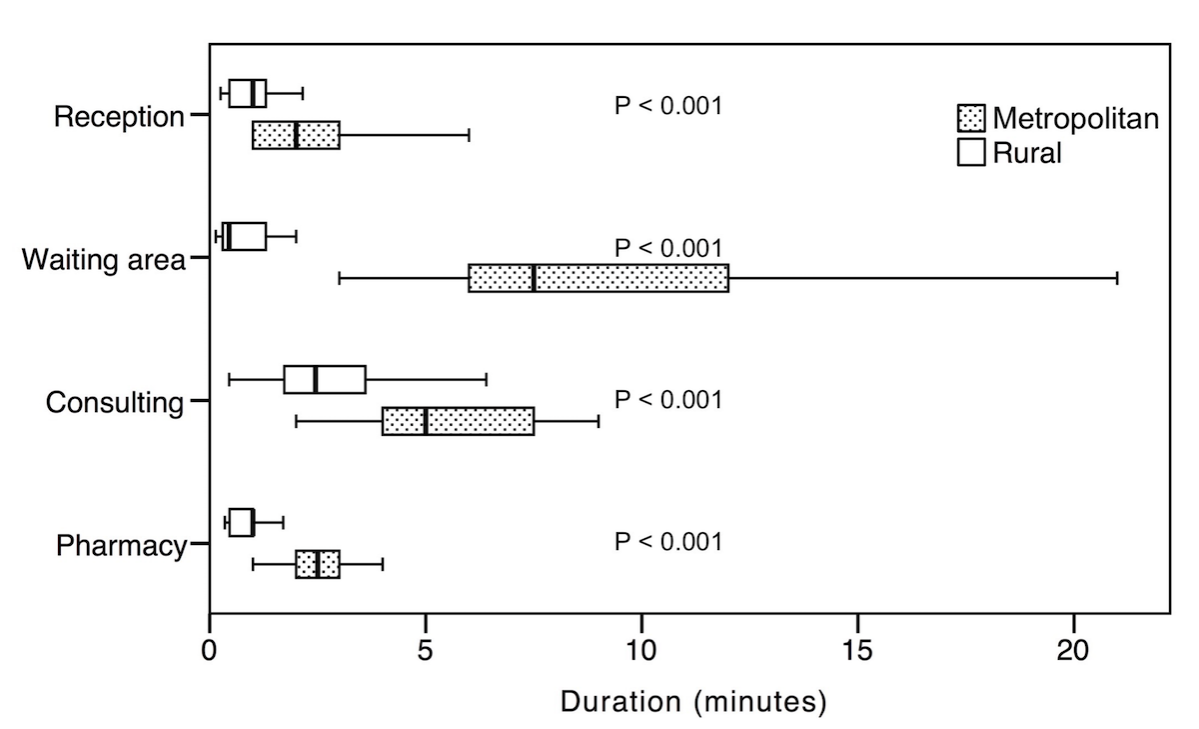
The time spent performing tasks at primary health care centers in metropolitan and rural areas.

The time spent performing tasks at each of the 4 PHC centers was also compared based on the location of the PHC centers (Figure 2). There were 2 metropolitan PHC centers and 2 rural PHC centers with 109 and 115 events, respectively. Our results showed statistically significant difference between the PHC centers located in metropolitan areas and the PHC centers located in rural areas for all 4 tasks, with *P<*.001 (Table 2).

**Table 2:** The time per task for primary health care centers located in metropolitan areas versus primary health care centers located in rural areas.

Task	Metropolitan		Rural		*P* value
	Number of events, n	Time (min), mean (SD)	Number of events, n	Time (min), mean (SD)	
Reception	30	2.36 (1.299)	32	1.09(0.710)	<.001
Waiting for doctor	26	9.73 (6.20)	25	2.07 (5.10)	<.001
Consultation time	23	7.43 (6.79)	31	2.83 (1.64)	<.001
Pharmacy	30	2.60 (1.22)	27	0.86 (0.42)	<.001

### Longitudinal Observation for Electronic Health Record–Based Primary Health Care Center

**Figure 3:**
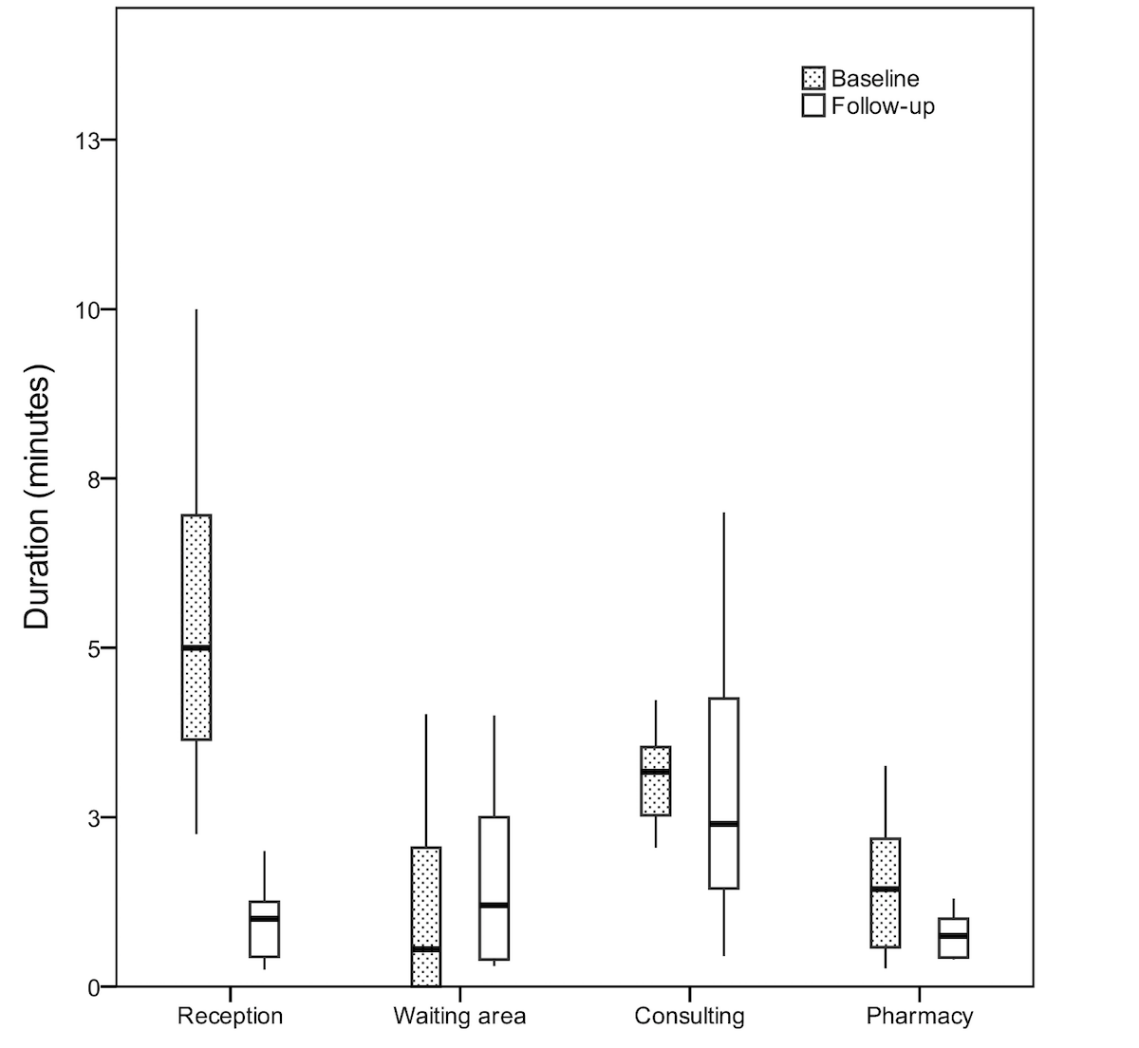
The time spent performing tasks at 1 electronic health record–based primary health care center after 1 year.

Finally, we examined the effect of familiarity with the EHR on the duration of tasks. At one of the EHR-based PHC centers, the observation was conducted longitudinally (Figure 3). There were 72 events at the baseline observation and 63 events at the 12-month follow-up. When comparing the time patients spent at each phase during their visits at baseline and follow-up, there was a significant difference in the time spent at the reception (*P<*.001) and at the pharmacy (*P*=.01). The difference in time patients spent waiting for the doctor and the consultation time was insignificant, with *P*=.22 and *P*=.36, respectively (Table 3).

**Table 3:** The time spent per task at baseline and 12-month follow-up at 1 electronic health record–based primary health care center.

Task	Before		After		*P* value	
	Number of events, n	Time (min), mean (SD)	Number of events, n	Time (min), mean (SD)		
Reception	15	5.5 (3.5)	16	0.9 (0.5)		<.001
Waiting for doctor	15	1.87 (3.5)	15	3.79 (7.89)		.22
Consultation time	20	3.1 (0.8)	18	3.05 (2.08)		.36
Pharmacy	22	1.49 (0.94)	14	0.75 (0.33)		.01

## Discussion

### Overview

The vast majority of prior research examined the impact of EHR on the duration of tasks from the user’s perspective (such as examining the time taken by doctors and other hospital staff to perform tasks). They often focus on a particular task or subtask that was aided by a new tool such as the computerized provider order entry or personal digital assistant. The impact of these tools on a patient’s time was rarely examined from the patient’s perspective [14]. In this study, we examined the difference in time taken per task for patients at PHC centers that used an EHR-based system compared with those that used a paper-based system. We also compared the time taken per task at PHC centers in the metropolitan and rural areas. Then, we examined the differences in task duration in 1 year and 2 years of EHR implementation.

One advantage of our study is that it controlled for many of the confounding variables that could vary based on the PHC centers. Some of these variables were the facility rules and regulations, funding, the type of system used (either EHR or paper form), and patient eligibility. We also applied the same data collection techniques using time and motion observations for both groups instead of using artifacts or a timestamp analysis.

Compared with prior studies, the average waiting time was relatively short. In a study conducted in the United States for instance, the average waiting time for a family physician was 13.5 min [23]. In our study, the average waiting time was 6.33, 5.47, 9.73, and 2.07 min for the PHC centers using an EHR-based system, using a paper-based system, in the metropolitan areas, and in the rural areas, respectively. Consistent with our result, a study conducted in Saudi reported that 83% of patients had a waiting time of less than 5 min [24].

Consistent with our findings, prior studies showed variations in the waiting time based on the geographical area [25]. Studies also reported that the distribution of PHC centers in the country was consistent with population distribution. This distribution resulted in overutilization of some PHC centers and underutilization of others [26]. The overutilization or underutilization of certain PHC centers could help explain why certain patients were unsatisfied with the waiting time at particular locations [8,20]. The concept of *satisfaction* was based on a self-administered survey, and the survey did not inquire about the exact waiting time and also did not provide documentation of the waiting time based on direct observations or EHR audit files [20].

Consultation time was an important factor as it impacts the quality of care, patient satisfaction, and level of utilization [18,27-29]. Although consultation time varies based on the country, studies showed that patients, in general, prefer a longer consultation time [18]. Studies based in the United States reported an average consultation time of 10 to 15 min, whereas a local study reported that 80% to 85% of patients spent less than 5 min with the doctor and 10% to 16% of patients spent 5 to 10 min with the doctor [24].

Comparing consultation times in this study with consultation times in prior studies must be done cautiously, as the local studies are outdated, and those that were conducted in the United States follow different workflow practices. In the United States, patients typically see a nurse who will take basic vital signs, collect medical history, and obtain general signs and symptoms [25]. Following this interaction with a nurse, patients will then wait at the doctor’s office to be seen [25]. This waiting time is sometimes counted as part of the consultation time, which will result in a longer patient-doctor interaction. In the PHC centers where our study was conducted, patients are called directly into the room to see the doctor, and the visit with the doctor begins at this point with no waiting time in between [25].

Consistent with prior studies, no significant difference was found in the duration of tasks between the PHC centers that use EHR-based systems and the PHC centers that use paper-based systems [12,17]. A significant difference was found in the duration of tasks between PHC centers based on location.

Our results did not show any significant difference in patients’ waiting time or consultation time after 2 years of EHR adoption. There was a significant difference in the time spent registering and at the pharmacy. The time spent at the reception decreased from an average of 5.5 min (SD 3.5) in January 2018 to an average of 0.9 min (SD 0.5) in December 2018. The decrease in time could be attributed to the MOH Web-based booking system, which was adopted between January 2018 and December 2018. The MOH Web-based booking system called Mawid was implemented to allow patients to book, cancel, or reschedule appointments while also allowing individuals to manage referral appointments [30]. This booking service was provided by the government as part of a larger initiative, which was intended to help to verify patient identities by linking patients to a national ID. The Web-based booking system required patients to enter the information needed by the registration office online before visiting the PHC center. Although this service was provided initially as an optional service, many PHC centers have made the service mandatory for accepting nonurgent patients.

### Limitations and Future Work

Although we tried to control for confounding variables such as the type of EHR system being used, facility rules and regulations, funding, and patient eligibility, we did not account for some factors that could impact the generalizability of our study. One of the factors was patients’ conditions and demographics. Patients with more complex diseases may require more time for consultation and data entry. In addition, the type of EHR system being used can impact the outcome. Although the system being used at PHC centers is provided and approved by MOH [22], the generalizability of our finding to PHC centers that use a different EHR system is unknown. Moreover, we were unable to determine the effect of system familiarity on the reception time for the longitudinal part of the study because of the introduction of the Web-based booking system after the baseline period. Another limitation is that we did not measure the interobserver reliability. However, all observers were similar in their academic qualification, experience, and training received before data collection. For future studies, we recommend controlling for patients’ conditions and reasons for visits because of their expected impact on the duration of visits. This will not only help in explaining the source of variation in time but also improve the generalizability of the results.

Our result shows a significant difference in the duration of tasks between metropolitan and rural PHC centers; however, the cause of these differences is yet unknown. This could be further investigated in future studies by including more PHC centers and examining the potential factors such as reason for visits, staffing, and variation in workflow and clinical practice. Moreover, we recommend examining the waiting time and consultation time in the different cities within the country. In addition, more studies are needed to examine the impact of EHR on the way patients spend their time when visiting the doctor in more busy environments.

### Conclusions

Our study showed that the time spent by patients on the various tasks during PHC center visits is the same at both EHR- and paper-based PHC centers. We also found that patients’ waiting time and consultation time were the same after 1 year and 2 years of EHR implementation. The registration time, however, decreased when comparing the time after 1 year with the time after 2 years of EHR implementation. We expect that the change was attributed to the Web-based booking systems rather than EHR itself. Apart from the training and skills related to short-term impact after EHR implementation, we believe that changes in time after EHR use are often attributed to the addition or elimination of tasks and functions rather than EHR itself. Therefore, focusing on the EHR function that minimizes the tasks performed by patients can shorten the duration of their visits and enhance their satisfaction. Some of these tasks include Web-based tools for booking, entry of patients’ history, and medication refill.

## Abbreviations

EHR: electronic health record
MOH: Ministry of Health
PHC: primary health care
